# THE SAAF STUDY: evaluation of the *Safeguarding Children Assessment and Analysis Framework* (SAAF), compared with management as usual, for improving outcomes for children and young people who have experienced, or are at risk of, maltreatment: study protocol for a randomised controlled trial

**DOI:** 10.1186/1745-6215-15-453

**Published:** 2014-11-20

**Authors:** Geraldine Macdonald, Jane Lewis, Kenneth Macdonald, Evie Gardner, Lynn Murphy, Catherine Adams, Deborah Ghate, Richard Cotmore, Jonathan Green

**Affiliations:** School of Sociology, Social Policy and Social Work, Queen’s University, University Road, Belfast, BT7 1NN UK; The Colebrooke Centre for Evidence and Implementation, 55 St John Street, London, EC1M 4AN UK; Nuffield College, Oxford, OX1 1NF UK; Northern Ireland Clinical Trials Unit, The Royal Hospitals, 1st Floor Elliot Dynes, Grosvenor Road, Belfast, BT12 6BA UK; Evaluation Department, National Society for the Prevention of Cruelty to Children (NSPCC), 42 Curtain Road, London, EC2A 3NH UK; Institute of Brain, Behaviour and Mental Health, Jean MacFarlane Building, Oxford Road, Manchester, M13 9PL UK

**Keywords:** Assessment, Decision-making, Child protection, Social work, Child maltreatment, Implementation

## Abstract

**Background:**

Serious case reviews and research studies have indicated weaknesses in risk assessments conducted by child protection social workers. Social workers are adept at gathering information but struggle with analysis and assessment of risk. The Department for Education wants to know if the use of a structured decision-making tool can improve child protection assessments of risk.

**Methods/design:**

This multi-site, cluster-randomised trial will assess the effectiveness of the *Safeguarding Children Assessment and Analysis Framework (SAAF*). This structured decision-making tool aims to improve social workers’ assessments of harm, of future risk and parents’ capacity to change. The comparison is management as usual.

Inclusion criteria: Children’s Services Departments (CSDs) in England willing to make relevant teams available to be randomised, and willing to meet the trial’s training and data collection requirements.

Exclusion criteria: CSDs where there were concerns about performance; where a major organisational restructuring was planned or under way; or where other risk assessment tools were in use.

Six CSDs are participating in this study. Social workers in the experimental arm will receive 2 days training in SAAF together with a range of support materials, and access to limited telephone consultation post-training.

The primary outcome is child maltreatment. This will be assessed using data collected nationally on two key performance indicators: the first is the number of children in a year who have been subject to a second Child Protection Plan (CPP); the second is the number of re-referrals of children because of related concerns about maltreatment.

Secondary outcomes are: i) the quality of assessments judged against a schedule of quality criteria and ii) the relationship between the three assessments required by the structured decision-making tool (level of harm, risk of (re)abuse and prospects for successful intervention).

**Discussion:**

This is the first study to examine the effectiveness of SAAF. It will contribute to a very limited literature on the contribution that structured decision-making tools can make to improving risk assessment and case planning in child protection and on what is involved in their effective implementation.

**Trial registration:**

ISRCTN 45137562 15 July 2014.

## Background

In 2010, Professor Eileen Munro was commissioned to chair a review of the child protection system in England. As part of a wide-ranging brief, she was charged with generating ideas about how to improve early intervention, enhance trust in frontline social workers and improve transparency and accountability in child protection. A central question for the review panel was ‘what helps professionals make the best judgments they can to protect a vulnerable child?’ [1, p.6]. The final report [1] highlighted the failure of historical attempts to improve assessment and decision-making via increased regulation, guidance and procedural requirements, rather than by developing and supporting the analytic and decision-making skills of social workers. It therefore recommended moving away from a culture of prescription and compliance (the ‘status quo’) to one that emphasised the importance of professional judgement. Achieving this necessitates ensuring that staff are equipped with the necessary knowledge and skills to exercise sound judgement, and chapter 6 of the *Final Report* addresses these issues in detail, noting the importance of the ‘ability to analyse critically the evidence about a child and family’s circumstances and to make well-evidenced decisions and recommendations, including when a child cannot remain living in their family either as a temporary or permanent arrangement; and skills in achieving some objectivity about what is happening in a child’s life and within their family, and assessing change over time’ (p.96).

There is a wide body of evidence to suggest that social workers are adept at gathering information, but find it challenging to analyse complex bodies of evidence and reach accurate judgement as to whether a child is suffering, or is likely to suffer, significant harm. Some studies have suggested that child protection assessments are ‘only slightly better than guessing’ [2]. Key reasons for poor quality assessments and decision-making are an inability or failure critically to appraise information collected, random errors, and our susceptibility to sources of bias such as ‘observation bias’ (a tendency to see things and people in a particular way, based either on what we are told about them beforehand or on the basis of certain features), ‘cultural relativism’ (the tendency to exercise different standards across different cultures) and the dominance of first impressions. These, and other sources of bias, have consistently been implicated in decision-making, as analysed by serious case reviews and inquiries into child deaths. Again, research suggests that providing professionals with tools to help them organise and critically appraise information in a systematic way can minimise bias and error and improve decision-making.

Structured decision-making (SDM) has been defined as a ‘general term for the carefully organized analysis of problems in order to reach decisions that are focused clearly on achieving fundamental objectives’. SDM draws both on decision theory and risk analyses and, in the field of child protection, has been described as ‘an example of an effort to integrate predictive (actuarial) and contextual assessment strategies’ [3]. So, for example, the Family Strengths and Needs Assessment (FSNA) is a structured approach to assessing (including scoring) child and family functioning in those domains recognised as important in child maltreatment - both from the point of view of causation (which actuarial tools do not address) and intervention. This aims to ensure a ‘logical fit’ between assessment and response.

The potential of structured approaches to improve assessment and decision-making has been reinforced by the findings of a systematic review of models of analysing significant harm, commissioned by the Department for Education (DfE) [4]. This review identified *two* SDM tools, both developed in the UK, which the review authors considered worth evaluating. Both address the three domains of the statutory guidance provided to professionals (known colloquially as ‘the Assessment Framework’) [5], namely, the child’s development needs; family and environmental factors, and parenting capacity.

The review authors note that both provide practitioners ‘with clear guidance about what to assess, and how to analyse and ‘make sense of’ the data collected’ (p.73). In relation to ‘Case Planning’, they note that only one of these - the Safeguarding Children Assessment and Analysis Framework (SAAF) - includes an assessment of the possibilities for future change (p.75). Based on this review, and evidence from the Munro report, the DfE commissioned this randomised trial of the effectiveness of the SAAF, alongside an implementation evaluation. Interventions such as SAAF are complex interventions, enacted in the complex environments of local authority (LA) Children’s Services Departments (CSDs). Understanding the process of implementation will inform the interpretation of the results of the trial and provide valuable learning to inform future use and roll-out.

### Safeguarding assessments

The work of social workers in CSDs is governed by legal statute. Section (S)17 of the Children Act 1989 lays upon them a ‘general duty … to safeguard and promote the welfare of children within their area who are in need’. Children are defined as being ‘in need’ when:

- (they are) ‘unlikely to achieve or maintain, or to have the opportunity of achieving or maintaining, a reasonable standard of health or development without the provision … of services by a local authority …’;
- (their) health or development is likely or be significantly impaired, or further impaired, without the provision of such services; or
- (they) are disabled

Children in need include children in need of protection, but S47 of the same Act lays down a specific duty to ‘make enquiries’ in respect of any child where there is ‘reasonable cause to suspect that … (he or she) … is suffering, or is likely to suffer, significant harm’. It is therefore commonplace to categorise referrals as either S17 (child in need) or S47 referrals.

Enquiries made under S47 are typically those where specific concerns have been raised about a child’s safety. However, a significant percentage of S17 cases may also raise concerns about a child’s development that are attributable to inadequate parenting and where assessment of family functioning is complex. For the purposes of this study, the impact of SAAF will be assessed with regard to complex assessments in both S17 and S47 cases.

### Aims of the study

#### Primary aim

The primary aim of this study is to determine whether complex assessments undertaken under S47 or S17 of the Children Act 1989 by social workers using SAAF, result in children being less likely to experience maltreatment or re-abuse than children whose social workers do not use SAAF.

It is hypothesised that social workers using SAAF will make more accurate assessments of risk and better child protection plans, including whether or not to remove a child from the care of his or her parents and the identification of effective intervention and protection plans to ensure their safety.

For the purposes of this study, complex assessments are those that require information to be gathered from a variety of sources in order to understand what is happening within a family, and where there are concerns about the adequacy of parenting, and/or whether a child has suffered, or is at risk of suffering, significant harm. Typically these assessments are referred to as ‘core assessments^a^’ or ‘comprehensive assessments’, although this language is now likely to change in the light of changes to guidance (see above).

#### Secondary aims

The secondary aims of the study are to determine the extent to which SAAF:

-  improves the quality of social work assessments of harm, future risk and parents’ capacity for change
-  is acceptable to social workers and other key stakeholders

If the data permit, the study will also seek to explore SAAF’s reliability in producing comparable assessment results across similar cases.

We will also seek to identify those implementation factors that hinder or facilitate its use and the reasons underpinning any adaptations made by individuals or teams.

### Study design

A multi-site, cluster-randomised controlled trial (RCT) in which teams of child protection social workers, stratified by site, are randomly allocated to one of two arms:

i.) SAAF training followed by implementation of SAAF in S47 cases and complex S17 cases;
ii.) Management as usual in S47 cases and complex S17 cases.

An implementation evaluation will run concurrently with the trial to explore the experience of using the SAAF, how it is integrated into working processes, and the barriers and facilitators to successful intervention. The study will also explore participant social workers’ experience of taking part in the trial.

## Methods

### Study sites

Six CSDs in England have been recruited by the DfE (Leeds, Oldham, Nottinghamshire, Greenwich, Bournemouth and Hampshire). These represent different CSD types (unitary, county, and metropolitan) located in a range of geographical areas (North West, Yorkshire and Humber, East Midlands, South East, South West and London).

### Eligibility criteria

#### Children’s services departments

##### Inclusion

CSDs in England were eligible for the study if they were willing to make relevant teams available to be randomised, willing to make staff in the experimental group available for training, and willing to require all participating social workers to comply with the study’s data requirements.

##### Exclusion

CSDs were not eligible if one or more of the following pertained: there were concerns about performance (for example, special measures, other DfE involvement), a major reorganisation was planned, the CSD was already using another risk assessment tool (for example, Graded Care Profile or Signs of Safety), the CSD had received training in recent years from the providers of the intervention, namely *Child and Family Training*.

#### Social work teams

Eligible teams within each CSD will be those that - between them - deal with the majority of complex S17 and S47 cases. Social workers in these teams are eligible, irrespective of experience or whether they are employees or agency staff. Generally, this will exclude teams that are working with looked-after children, court-work teams (where decisions have already been made that the level of risk posed to children justified their removal from parents’ care), intake teams or Multi-Agency Safeguarding Hub (MASH) teams (‘single point of entry’ teams who largely act as conduits to other teams’ services).

### Intervention and comparison

#### Experimental group: Safeguarding Children Assessment and Analysis Framework (SAAF)

The SAAF assessment tool asks social workers to make a judgement relating to each of 55 items that social workers should consider when making their assessments:

-  For 33 items these are judgements of level of risk or concern (both terms are used), covering the child’s developmental needs, parenting capacity and family and environmental factors. Whilst explicitly not a score card, social workers are asked to rate each item on a five-point Likert-type scale, one end of which represents ‘low level of concern’ and the other ‘high level of concern’.
-  For 22 items these are judgements about prospects for intervention, covering parenting capacity, family and environmental factors, and child’s developmental needs. Again, this is not a score card. Social workers are asked to indicate where, again on a five-point scale, they judge the prospects for (successful) intervention to lie, with ‘reasonable prospects of success’ at one end, and ‘poor prospects’ at the other.

Social workers are then asked to make three summative judgements, using a three-point scale:

- Level of harm (low, moderate, high);
- Level of risks of re-abuse or likelihood of future harm (low, moderate, high level of risks); and
- Prospects for successful intervention (poor, moderate, better prospects).

This is then used as a basis for guiding decision-making.

The SAAF approach to assessment and analysis will be taught to social workers in the experimental arm in a two-day training course by SAAF’s developers - *Children and Families Training*. Social workers who attend SAAF training will receive the following:

-  A two-day training course aimed at improving understanding of how best to approach the task of complex assessments. The training includes:
  - helping social workers to distinguish between the *collection* of relevant information on each of three assessment domains (Child's Developmental Needs, Parenting Capacity, and Family and Environmental Factors) and *hypothesising* how particular data might be related
  - instructing social workers on the use of a series of grids to structure and critically appraise information, with particular reference to estimating the risk to the child if nothing is done, what needs to change in order to safeguard the child, and what interventions are best placed to achieve those outcomes, and estimates of parents’ capacity to change and their willingness to engage with an appropriate protection plan
-  Materials to further develop their competence, and support their use of the SDM tool including:
  - ▪ SAAF User Guide
  - ▪ SAAF Instruments Record
  - ▪ A book about SAAF produced by its developers [6], and
  - ▪ Access to resources on Children and Family Training’s website
-  SAAF grids, transposed from paper to an electronic format to facilitate their use
-  Limited post-training telephone consultancy, delivered by the trainers (Child and Family Training) to discuss problems and issues that might have emerged

The SAAF tools and training are designed to improve the *quality* of the assessments produced, and not to replace policies, practices or proformas already in use within the participating departments. Social workers using SAAF may append or use information from additional tools or sources of information, but they will continue to use the forms required by their employer, and adhere to any other policy or procedure.

#### Control group

‘No treatment’ is not an option when children are referred to CSDs because of concerns about risk of harm or inadequate parenting. The control condition will therefore be ‘management as usual’. Social workers in the control arm will continue to follow departmental policy and undertake S47 enquiries and complex assessments associated with both S47 and S17 (Assessment of Children in Need) cases, developing Child Protection Plans (CPP) as usual, supported by relevant policy guidance and management systems.

### Outcomes

#### Primary outcome

Differences between the two arms in the proportion of cases resulting in maltreatment or recurrence of maltreatment following the completion of an assessment (S17 cases) or initial child protection conference (S47 cases).

*Measures*: using administrative data (CIN data^b^) collected by LA CSDs we will assess the (re)occurrence of maltreatment, as defined by:

-  Number of children who become subject to a CPP for a second or subsequent time (or for the first time following a S47 or S17 assessment that did not result in a CPP), as a result of concerns linked to the original assessment;
-  Number of reassessments or re-referrals as a result of concerns linked to the original maltreatment/perceived risk of maltreatment;

At a national level, the CIN data include items such as ‘Initial category of abuse’ and ‘Latest category of abuse’. In order to determine whether or not the trigger incidents are related, that is are indicative of a failed plan or inadequate assessment, data are needed that provide information at a more granular level than that typically collected for the National Statistics Office (the annual CIN return^c,d^). For this purpose we will use the more detailed data gathered by the CSDs (information management) and collect data immediately post-assessment from social workers *via* an electronic questionnaire (the Case Report Form). These data will provide us with information about the concerns of social workers, their confidence in their assessments, their plans and assessments of future risk. They will also ensure that i) we do not miscategorise apparently unconnected events that in fact have a common underlying cause. For example, physical abuse by a parent and sexual abuse by a stranger *may* be unrelated, but they may also be symptoms of a seriously neglectful environment; ii) we can link children who move between one form of assessment or focus to another; for example, S17 to S47, and monitor associated changes in assessment.

#### Secondary outcomes

1. Quality of assessments undertaken using SAAF:

High quality assessments are necessary but not sufficient for minimising the chances of (repeat) maltreatment. Other factors, such as missing information (that could not have been available to the social worker), changes in circumstances, the lack of appropriate services, or disagreement amongst professionals, may result in future maltreatment following an assessment that a child is not in need of protection (S17) or the implementation of a CPP (S47 cases). An assessment of the impact of SAAF on the quality of assessments, independently of outcomes, is therefore included in this study.

When 1,800 complex assessments have been completed, the Clinical Trials Unit will randomly select 10% of assessments, stratified by study arm and size of CSD. These 180 assessments will then be quality assessed by members of the research team, blind to whether the assessment is from the experimental or control arm of the study. It is not possible to blind the assessors to CSD given the forms used in each department. The researchers will be asked to record any information that might lead them to believe they know the arm from which the assessment was drawn; for example, reference to SAAF.

*Measures*:

-  Data relating to the quality of assessments will be recorded using a quality assessment schedule developed for this study. This requires the assessor to collect information on 44 items related to assessment quality.
   For 30 items the responses are simply ‘yes’ (score 1) or ‘no’ (score 0). For example, item 27 asks: ‘Does the assessment make clear the changes required in the child’s care to make the child/ren safe?’, and the responses open to the assessor are: ‘Yes, the assessment makes clear the changes required in the child’s care to provide them with adequate parenting’ or ‘No, the assessment fails to makes clear the changes required in the child’s care to provide them with adequate parenting’.
   For the remaining 14 items there are 3 possible responses, reflecting the factor being assessed; for example, item 24 asks: ‘If included, is there evidence that, in reaching their problem formulation, the author considered other, plausible explanations?’ and the assessor is asked to select from the following 3 responses, scored respectively 2,1,0: ‘The assessment provides evidence that the social worker considered alternative theories that might explain how the present situation has come about, and has provided reasons why s/he favours the one put forward’; ‘The assessment provides no evidence that the social worker considered alternative theories that might explain how the present situation has come about, but s/he provides reasons why/evidence for the hypotheses being proposed’; ‘The assessment provides no evidence that the social worker considered alternative theories that might explain how the present situation has come about, and no reason/evidence for the hypotheses being proposed’. The maximum possible score for any assessment is 48.
   This is not a validated tool, but it is based on factors known to be associated with quality assessments. Whilst SAAF is designed to improve assessment and analysis, the tool is not biased towards the content of SAAF. Researchers will be provided with a user guide, which provides guidance on what is being looked for, and how to score items. For example, in relation to item 24 (above) about problem-formulation, the user guide states: ‘*Research indicates that premature conclusions can lead to mistakes, some of which can be fatal. It is good practice to consider alternate explanations or theories, and to be able to articulate why one has opted for one particular explanation theory, rather than another*’. The schedule will be piloted, the findings discussed, and re-piloted, until a satisfactory rate of inter-rater reliability is achieved. Assessors will receive training in the tool, and will be required to attain a satisfactory reliability rating before assessing SAAF and control assessments.
-  Information gathered from social workers on their approach to assessment, information collected and their confidence in the assessment and, where relevant, the proposedCPP.

2. Relationships between SAAF assessment judgements (55), overall assessments (3) and child protection plans/interventions.

In the review that identified SAAF as a promising tool to improve social work safeguarding assessments [4], the authors emphasise the importance of assessing the reliability and validity of the SAAF as a ‘tool’ to improve the classification of risk and the development availability of evidence-based programmes for those families assessed using SAAF.

We cannot directly address issues of inter-rater reliability within the resource constraints of the current project, but we will investigate:

-  the extent to which the structured approach (55 judgements) is linked to the 3 summative assessments of harm, risk and prospects for intervention; to recorded variations in CPPs, and to the primary outcome.
-  the extent to which the three summary judgements are linked with subsequent maltreatment or its absence.

Data relating to the 55 judgements and 3 summative judgements used in SAAF will be obtained directly from the SAAF forms used by social workers in the experimental arm, and from the Case Report Forms.

### Timeframe

Primary outcomes will be assessed at 6 and 12 months after the completion of an assessment.

Assessment quality will be assessed once the social worker’s assessment has been signed off by the relevant line manager. The relationship between SAAF assessment judgements, overall assessments and CPPs will be undertaken when data are available on all assessments included in the trial, together with analyses of the relationship between the three summary judgements and subsequent maltreatment.

#### Intervening variables

The impact of SAAF on the recurrence of abuse is likely to be mediated by factors that *intervene* between a social worker assessing a family and what happens to that child and family some 6- or 12-months later. Social workers might not feel they have sufficient time to conduct their assessment properly, whether or not they are using SAAF; other professionals may disagree with their assessments, or their assessments may point to interventions that are effective, but unavailable.

We will collect information on a range of potential *intervening variables* as part of our implementation evaluation, and from social workers at the end of each assessment. We will also collect information on the influence that social workers perceive their assessments to have on the decisions of Child Protection Case Conferences (CPCC), including the attention paid to their assessment of risk and the child protection plan/profile of services provided. These data will be sourced from social workers at the end of each assessment, from the SAAF tools (see above), and interviews with child protection chairs and independent reviewing officers.

### Ethical issues

The study was granted ethical approval by the Ethics Committee of the School of Sociology, Social Policy and Social Work, Queens University, on 16 May 2014 (Ref: *EC/167)*.

CSDs are consenting to participate in this study, and have written to the DfE confirming this. CSDs have subsequently confirmed their consent to participate to the principal investigator (PI). All social workers based in the selected teams will be participating as part of their employment. All social workers participating in this study will attend a briefing session with the PI, trial manager, and representatives from DfE and *Child and Family Training*. At this session, the social workers will be given further information about the purpose of the study and their role within it and will be given the chance to ask questions. They will also be provided with a participant information sheet (available from the PI).

On advice from the DfE, consent is not being sought from parents. This is because the focus of the study is the quality of work undertaken by social workers.

### Study timeline

**Figure 1:**
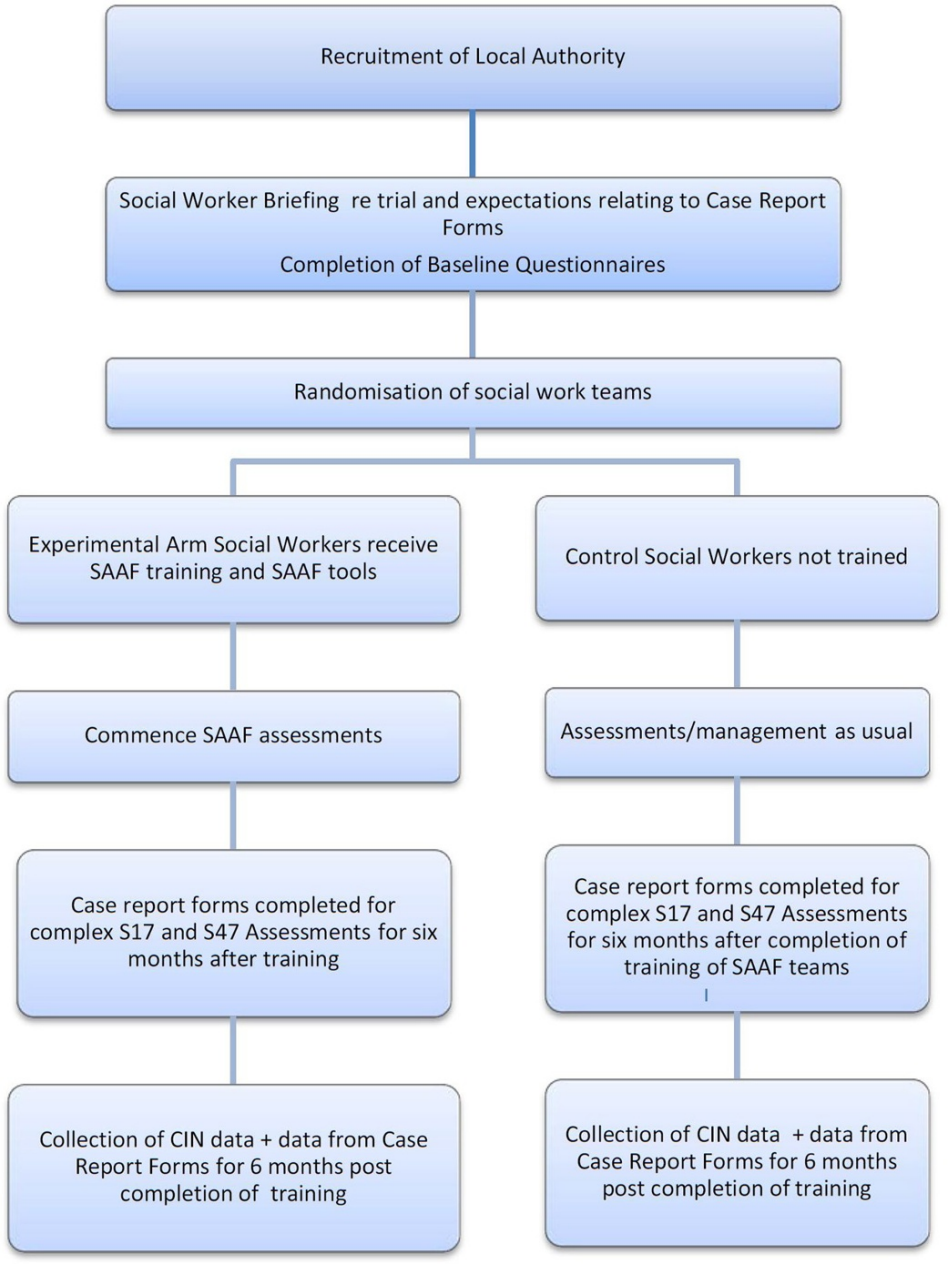
**Flow of cases/Safeguarding Children Assessment and Analysis Framework** (**SAAF) trained social workers within each Children’s Services Department (CSD).**

The trial formally commenced on 2 January 2014 (contract agreed). Recruitment by the DfE took place between December 2013 and April 2014.Following a study briefing session, social work teams in each of the participating CSDs will be randomised between May and August 2014. Social workers in the experimental arm in each CSD will receive training in SAAF in groups of 20. Once training is complete in a CSD, all social workers in that CSD will be required to provide information on each of the assessments they complete for a period of 6 months. The flow of work is not always predictable, and this period may be extended in order to obtain the necessary number of cases needed, or foreshortened in the even that this target is reached sooner. Figure 1 provides details of the timeline for each participating CSD.

### Sample size

The sample size was calculated using one of the two measures of the primary outcome - maltreatment, repeat CPPs. Of the two measures (the other being re-referral), this was judged to be the most valid measure of re-abuse within the Children in Need (CIN) Database, the source specified by the funder, the DfE. Re-referrals include large percentages of children in need, but not in need of protection; for example, day care, play groups, family support. Data on these indicators can be found in SFR45-2013^b^.

Children who are deemed in need of protection (that is, are assessed as at risk for significant harm) are subject to a CPP. This sets out what is needed to protect the child and to address the causes of significant harm or risk of significant harm. A successful CPP should remove the threat of significant harm, or reduce it to a threshold that professionals believe can be managed without a CPP. Children who are subject to a second CPP are likely to have been subject to repeat maltreatment, or to be children for whom concerns have increased following the first CPP. The need for a second CPP (or a CPP following an assessment that has judged a child not to require one in the first instance) may indicate a poor quality assessment.

A number of factors make it difficult to arrive at precise power calculations, namely:

-  the lack of an available inter-cluster correlation (ICC) for this purpose;
-  variation in size of clusters (range 6 to 12);
-  different rates of CPPs amongst participating CSDs (range 13.2% to 17.5%);
-  inability to estimate re-referrals and repeat CPPs within the time frame of the study; for example, amongst repeat CPCCs, there are no data on time to repeat CPCC, and most CSDs indicated that this would be rare ‘within the year’^e^, and
-  the complexity of the relationship between S17 and S47 assessments, given that one case may have recorded 2 separate assessments.

Together, in the year ending 31 March 2013, the 6 participating CSDs undertook: 8,524 S47 enquiries; 16,395 core assessments^a^ and 5,394 CPCCs.

Table 1 documents a range of scenarios reflecting various postulated rates of re-abuse in the control group, and varying the design effect (variance inflation factor) from 1.5 (based on an estimated ICC of 0.1 and a cluster size of 6 social workers *per* team) to 2 (which allows, with the same ICC, roughly for an increase in cluster size to 10 to 12 social workers *per* team).

**Table 1:** **Scenario planning - power calculations undertaken to inform the sample size**

Scenario	Re-abuse Rate % Control	Re-abuse Rate % SAAF	Unclustered total sample size	Design effect	Clustered total sample size (rounded)
1	50	40	800	1.5	1,200
2a	25	15	510	1.5	770
2b				2.0	1,000
3a	10	5	900	1.5	1,300
3b				2.0	1,800
4a	10	2.5	400	1.5	600
4b				2.0	800
5a	20	10	400	1.5	600
5b				2.0	800

Given the uncertainties in the CIN data, and in the absence of an existing, secure ICC from other studies, we judged that a design effect of 2.0 was most appropriate given the rate of repeat CPPs. This points to the need for an achieved sample size of 1,800 cases, which we estimate will be achieved within a 6-month period following the training of the experimental social workers in each CSD.

The design of the study, including reliance on administrative data, attenuates the need to factor dropout into the calculations.

### Recruitment

The six sites listed above were recruited by the DfE, in consultation with the study team. A total of 19 CSDs were approached by the DfE and the 6 included sites were those that expressed an interest and had capacity.

### Assignment of interventions

- *Sequence generation*: social work teams within participating LAs will be randomly allocated to one of the two study arms. The allocation will be achieved by computer generated random numbers by the Northern Ireland Clinical Trials Unit (NI CTU) using randomly permuted blocks.
- *Allocation concealment*: the NI CTU will inform the PI and Trial Manager of the allocation of each social work team. The Trial Manager will then Email the designated contact person in each CSD to inform them of their allocation.
  The Trial Manager will also inform Children and Family Training, who will then liaise directly with the CSD to arrange training for those social workers/teams in the experimental arm.
- *Implementation*: the allocation to the intervention or control arm will be generated by the NI CTU and conveyed securely to the Trial Manager and PI.
- *Check on baseline equivalence*: all social workers will complete a baseline questionnaire prior to randomisation, which will provide information on their experience, training to date, expectations of the training, attitudes towards the trial, and so on. This will provide some indication of baseline equivalence between the two arms, as well as informing the interpretation of the study results.
- *Blinding*: given the nature of the intervention, the data to be collected, and the interface with the Trial Manager (who will be the point of contact for enquiries regarding data collection), it will not be possible to maintain the concealment of allocation, that is social workers will know whether or not they have been trained. Further, the Case Report Form will include questions about the use of the SDM tools (which the control group social workers will not be using) and the assessments that will be quality assessed may include indications that the authors were in receipt of training/used the SAAF SDM tools.

However, we will endeavour to select assessments for audit without divulging the arm from which they were sourced to those conducting the quality assessment. Each CSD will be asked to provide between 25 and 40 assessments, randomly selected by the NI CTU, from participating social workers in each arm, and make these available to the researchers undertaking the quality audit, without divulging which teams the authors belong to.

We will also include a question on the quality assessment schedule that will enable assessors to indicate whether or not the assessment led them to believe it had been undertaken by a social worker in the SAAF (experimental) arm.

#### Implementation evaluation

Given that this is a complex intervention, we are conducting an implementation evaluation alongside the effectiveness study, informed by the emerging field of implementation science [7–10]. We will conduct an online survey of social workers in the experimental arm to explore whether SAAF was implemented as intended, their perceptions of SAAF, how easy it was to use, how relevant and useful. We will explore the extent to which staff felt sufficiently skilled to learn and use SAAF, whether and how it was embedded in working practice, the processes and resources they feel were necessary for its successful implementation, including buy-in from managers and other key stakeholders (and whether any of these were not in fact in place). These data will also be used to explore the possible reasons for differences across the participating CSDs. The online survey will be supplemented with a number of in-depth interviews with key stakeholders from each CSD to explore implementation in more detail. Control group social workers will also be surveyed by means of a short, on-line questionnaire to ascertain detailed information about management as usual.

In order to explore the perceived impact of training, social workers in the experimental group will be asked to complete a second questionnaire following the SAAF training to explore its impact on their perceived knowledge and skills in assessment.

#### Data on baseline equivalence

In order to explore the extent to which randomisation has created two equal groups, the study will collect relevant data from participating social workers on their qualifications, experience and confidence in relation to complex assessments, and knowledge in relation to key areas (for example, mental illness, intimate partner violence, substance misuse). These data will be collected from all social workers following a study briefing.

### Data management

Case Report Forms will be completed on-line by social workers, using a unique identifier for both the social worker and the family. All data will be stored securely and no identifying details of the family/child will be recorded.

The DfE will flag each child whose assessment is used in the trial to facilitate later follow up, should funding be available. These data will remain anonymised (that is no child’s name would be used).

All data will be monitored using central statistical monitoring for consistency, viability and quality.

Data from the study will only be presented in public once the main results are published in peer reviewed journals according to Consolidated Standards of Reporting Trials (CONSORT) guidelines and disseminated to all the study participants (CSDs) in an accessible format.

### Data analysis

#### Assessing trial validity

Initial data analysis (descriptive statistics and bivariate tests) will examine the extent to which the necessary conditions required to permit a valid test of the efficacy of SAAF have been met [11]. This will include assessment of the achieved statistical power, patterns of attrition (social workers leaving/moving), between-group equivalence on key factors (for example, staff turnover, team size, experience of social workers, types of cases), SAAF fidelity (the extent to which SAAF appears to have been used as intended) and discriminability (for example, the approach to assessment undertaken by control social workers is sufficiently distinct).

#### Assessing the effectiveness of SAAF

##### Intention-to-treat analyses

The primary outcome analysis will be an intention-to-treat analysis (ITT) such that all cases will be assessed in accordance with the randomisation. Analysis will be conducted both within and across LAs. We will maximise use of administrative data in order to document the extent of differences between the experimental and control groups.

##### Outcome measures

Some outcome measures are binary and some are continuous. Estimation methods will vary depending on whether the dependent variable is binary or continuous, but the logic of the analysis will in each case be the same.

##### Inclusion of covariates

In addition to the standard ITT, multivariate (ordinary least squares (OLS) and logistic regression) models will be estimated to examine the impact of covariates on outcomes. Baseline outcome measures (for example, type of abuse, risk factors identified) will be included as covariates to allow for individual differences, and site differences will be modelled. Including information on covariates will allow us to examine moderator effects and to begin to unpick the mechanisms through which SAFF might impact on improved assessments and associated outcomes.

A key part of this analysis will be to try to minimise the unexplained variance in site-specific effects. This will increase power and, by capturing the factors that explain why effects vary across sites, will help in generalising the results beyond the study sites. Thus, we will look at possible sources of variation across sites - in participant characteristics, in staff experience and in what constitutes management as usual; for example, including site-specific averages as controls in regression analysis.

##### Multilevel models

If appropriate, we will also employ nested modelling techniques for random effects models (such as ML-win), as well as comparing the results with a fixed-effect model. Multilevel logistic regression models will be used to assess between group differences (experimental and control) in relation to the probability of abuse recurrence for cases, accounting for the fact that cases are clustered within social work teams.

Ancillary analyses will assess the extent to which:

-  rates of abuse recurrence vary according to: rates of staff turnover, severity of initial maltreatment, and
-  the presence of SAAF trained social workers in the control arm affects rates of recurrence (with similar analyses conducted on the effect of untrained workers on experimental group).

##### Treatment of missing data

Examination of missing data (both case and item) will be undertaken on outcome measures and covariates. Depending on the result of this, multiple imputation methods [12, 13] may be employed to reduce biases due to any missing responses within the ITT analysis [14]. Consideration will also be given, where appropriate, to modelling strategies that generate robust standard errors in the presence of missing data (that is Full Information Maximum Likelihood; FIML). The greater reliance on administrative data, where sample members can be tracked irrespective of attrition or compliance in the trial, will provide very valuable information on non-missing at random for attrition, and hence greatly improve the accuracy of multiple imputation.

##### Sensitivity analysis

Analysis will be undertaken to assess the robustness of the outcome analysis. This will include the repetition of the analysis on alternative specifications of outcome measures, different subsets of the study population (that is per protocol analysis), and with different missing data models.

### Harms

No direct client contact is planned, and there is no reason to believe that providing social workers with additional training in analysis and case planning will lead to deterioration in the quality of decision-making. However, we will monitor the data collected to ensure that any indication of poorer performance of the experimental group is identified and considered.

## Discussion

Staff in children’s social care in England have limited understanding of randomised trials, or experiencing of participating in them. The study was commissioned by the government department responsible for children’s social care; they are keen to adopt robust standards of evidence, but are relatively inexperienced in commissioning randomised trials, one consequence of which is that the timetable set has proved extremely challenging; for example, the need to develop the logic model underpinning the SAAF had not been identified at the commissioning, resulting in subsequent delays to the original timetable. Whilst the source of primary outcome data is administrative, important information is required from the participating social workers, who already feel overstretched. We have sought to minimise all data requests, and briefing sessions are being provided for all teams, to ensure that they understand the purpose of the trial, the implications of randomisation (and why it is important) and what we are asking of them. One of the challenges inherent in this trial is that staff turnover is significant in most CSDs, and many rely on agency staff, but those are the realities of children’s social care at the present time. This is the first study to examine the effectiveness of SAAF. It will add to a currently very limited literature on the contribution that structured decision-making tools can make to improving risk assessment and case planning in child protection and on what is involved in their effective implementation.

### Trial status at publication

Social work teams in all CSDs have been randomised. Training of staff in the experimental arm will be completed by the end of January 2015. Data collection relating to primary and secondary outcomes began in October 2014 in one of the six participating CSDs.

### Endnotes

^a^This is the term used for the full assessment (conducted within 35 working days) that followed an initial assessment (conducted within the first 10 days following referral). Following the Munro review, CSDs are in the process of transitioning towards a system whereby social workers have 30 days to produce full assessments. Assessments that require this time are typically those that are complex or detailed. Assessments that take little time to process are referred to as ‘straightforward’ in this study, and are not the focus of the intervention.

^b^SFR45-2013 [https://www.gov.uk/government/statistics/characteristics-of-children-in-need-in-england-2012-to-2013].

^c^[https://www.gov.uk/government/uploads/system/uploads/attachment_data/file/299928/DFE-00338-2014.pdf].

^d^[https://www.gov.uk/government/uploads/system/uploads/attachment_data/file/252790/Additional_guide_on_the_factors_CIN_Census_2014-15.pdf].

^e^The CIN data are generally not transparent to interpret; for example, the data on the category ‘re-referrals’ suggests that ‘referrals’ themselves contain a number of ‘re-referrals’.

## Abbreviations

CIN: Children in Need
CONSORT: Consolidated Standards of Reporting Trials
CP: child protection
CPP: Child Protection Plan
CPCC: Child Protection Case Conference
CSD: Children’s Services Department
CTU: Clinical Trials Unit
DfE: Department for Education
FIML: Full Information Maximum Likelihood
FSNA: Family Strengths and Needs Assessment
GCP: Good clinical practice
ISRCTN: International Standard Randomised Controlled Trial Number
ITT: intention-to-treat analysis
LA: local authority
MASH: Multi Agency Safeguarding Hub
NI CTU: Northern Ireland Clinical Trials Unit
OLS: ordinary least squares
PI: principal investigator
RCT: randomised controlled trial
S17: Section 17 (of the Children Act 1989)
S47: Section 47 (of the Children Act 1989)
SAAF: Safeguarding Children Assessment and Analysis Framework
SDM: Structured Decision-Making
TM: Trial Manager
TMG: Trials Management Group
TSC: Trial Steering Committee
UK: United Kingdom.
